# Utility of retesting for diagnosis of SARS-CoV-2/COVID-19 in hospitalized patients: Impact of the interval between tests

**DOI:** 10.1017/ice.2020.224

**Published:** 2020-05-11

**Authors:** Michelle E. Doll, Rachel Pryor, Dorothy Mackey, Christopher D. Doern, Alexandra Bryson, Pamela Bailey, Kaila Cooper, Emily Godbout, Michael P. Stevens, Gonzalo Bearman

**Affiliations:** Virginia Commonwealth University, Richmond, Virginia

Molecular testing of nasopharyngeal specimens for SARS-CoV-2 are highly specific and sensitive.^1,2^ However, SARS-CoV-2 viral shedding within the respiratory specimens of individual patients may not be dependable or consistent throughout the course of illness.^2-5^ The range of clinical presentations of COVID-19 present a diagnostic dilemma; reports of false positives^6^ add to uncertainty. Retesting of patients is increasingly requested in the setting of ongoing concern for COVID-19 after an initial negative test. Which patients should be prioritized for retesting and at what time interval are currently unclear.

## Methods

All patients admitted to a tertiary medical center with clinical concern for COVID-19 were referred to a team of infectious disease physicians for case review and testing approval. Retesting requests were largely driven by primary team concerns for false-negative initial test results. To avoid patients going off and back on isolation, an early interval retesting protocol was developed in which patients were held on isolation and retested 24 hours after the first result if they were categorized with high probability for COVID-19. Infectious disease physicians designated each patient with high or low probability based on the following clinical criteria consistent with reported literature^7^: (1) exposure to SARS-CoV-2; (2) symptoms of COVID-19, including hypoxia, respiratory or gastrointestinal symptoms, or fever; (3) leukopenia; (4) chest imaging; (5) lack of other explanatory diagnosis. Patients labeled with high probability who tested negative were held on isolation another 24 hours for retesting. Longer-interval retesting outside this protocol continued concurrently; providers could request retesting any time during the hospitalization. If approval was granted, these patients were reisolated for possible COVID-19 pending the repeat testing.

Nasopharyngeal specimens were collected by nurses who had received online training in specimen collection. On March 26, 2020, a patient tested negative on admission to our institution, but subsequently a previously collected outpatient test was positive. The resulting concerns about proper specimen collection were addressed by requiring nurses to do in-person retraining in a “train-the-trainer” model. Testing was performed using an in-house RT-PCR test developed from the Centers for Disease Control and Prevention (CDC) primers.

## Results

Overall, 70 inpatients with initially negative SARS-CoV-2 testing underwent repeat testing for ongoing clinical concerns between March 2 and April 4, 2020. One patient converted to a positive test; the interval between tests for this individual was 6 days. All other patients remained negative on repeat testing.

Early interval retesting of patients with a high pretest probability for SARS-CoV-2 as part of a formal protocol was performed from March 31, 2020, through April 7, 2020. During this period, 38 patients were deemed “high probability” by infectious diseases physicians using the standard criteria. Of the 38 patients with high pretest probability for COVID-19, 19 tested positive and 19 tested negative. The 19 “high probability” but negative RT-PCR patients were then re-tested within 24 hours and all remained negative. This protocol was abandoned after April 7, 2020, given a lack of observed clinical utility.

**Fig. 1.**
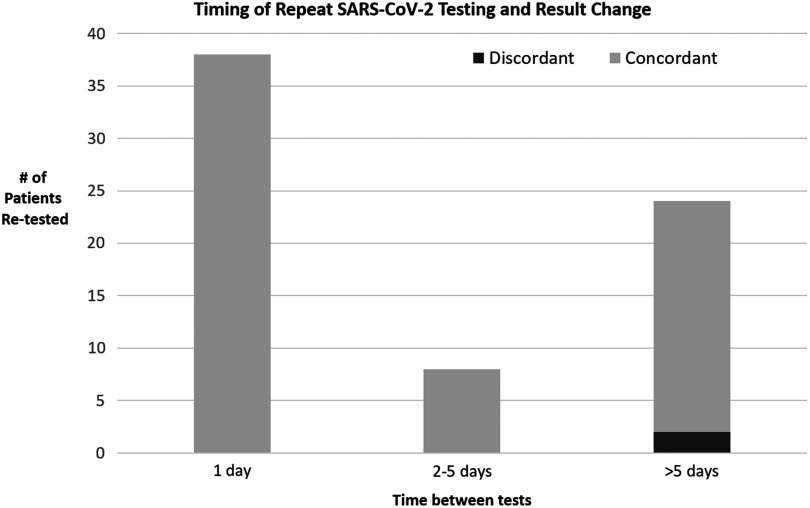
Timing of repeat testing and result change. Initially negative results were repeated for 70 patients. Concordant tests indicate patient remained negative on the second test. One patient had discordant results on repeat testing, becoming positive for SARS-CoV-2. All tests were performed using reverse-transcriptase polymerase chain reaction (RT-PCR) testing on nasopharyngeal swab upper respiratory specimens.

Overall, repeat testing was performed within 24 hours for 28 of 70 patients with no discordant results observed. Intervals between testing and result outcomes are shown in Figure 1. The patient who tested positive 6 days after a negative result was deemed “low probability” when re-evaluated for that repeat test.

## Discussion

Decisions to isolate and test inpatients for COVID-19 are balanced between concerns for overtesting or overuse of scarce PPE and undertesting with cross-transmission risks. Provider distrust of test results further complicates testing considerations.

Reports of serial patient testing indicate that the quantity of virus is highest in the first week after symptom onset, with a potential to decrease as patients recover.^3,4^ However, cases of high probability symptomatic patients with false-negative testing early in the course of illness have been reported.^5,6^ For example, Xu et al^5^ reported 3 patients presenting with respiratory illness in the setting of known exposures to SARS-CoV-2 who initially tested negative. Interval computed tomography (CT) scans over the next 1–2 days revealed findings concerning for viral pneumonia. Patients were retested, and the results were positive at an interval of 1–3 days.^6^ In a larger cohort, 258 patients were retested, and 15 converted from initially negative to positive results.^5^ The mean interval between these tests was 5.1 days (SD, 1.5 days; range, 4–8 days).^5^ Differences in testing platforms and specimen types should be taken into consideration; the CDC recommends nasopharyngeal samples as the preferred specimen type.^8^ Experience with repeat testing using samples obtained by nasopharyngeal sampling is lacking at present.

Our data suggest that short-interval testing is low yield. Assuming that specimen collection is appropriate, the presence or absence of virus in the nasopharynx or other sites is not expected to change dramatically within 24 hours. Our patient with discordant results in the course of symptomatic illness had testing performed at an interval of 6 days, suggesting that changes in viral shedding may have occurred over that time period.

Overall, our experience inspires confidence in the accuracy of the test. However, false negatives can occur for a variety of reasons. A better understanding of host factors associated with false negatives and/or decreased viral shedding while symptomatic is urgently needed to inform testing, retesting, and patient isolation protocols. Testing strategies incorporating samples from multiple sites, or other combinations of multiple test types,^9^ may become standard practice as validation continues. In the meantime, COVID-19 diagnostic uncertainty remains problematic for infection control and occupational health efforts.

## References

[1] Nalia AK , Casto AM , Huang MW , Perchetti GA , Sampoleo R , et al. Comparative performance of SARS-CoV-2 detection assays using seven different primer/probe sets and one assay kit. J Clin Micro 2020 [Epub ahead of print]. doi: 10.1128/JCM.00557-20. PMC7269385 32269100

[2] Tang Y-W , Schmitz JE , Persing DH , Stratton CW . The laboratory diagnosis of COVID-19 infection: Current issues and challenges. J Clin Micro 2020 [Epub ahead of print]. doi: 10.1128/JCM.00512-20. PMC7269383 32245835

[3] To KK-W , Tsang OT-Y , Leung W-S , et al. Temporal profiles of viral load in posterior oropharyngeal saliva samples and serum antibody responses during infection by SARS-CoV-2: an observational cohort study. Lancet Infect Dis 2020;3099:1–10. doi: 10.1016/s1473-3099(20)30196-1. PMC7158907 32213337

[4] Wölfel R , Corman VM , Guggemos W , et al. Virological assessment of hospitalized patients with COVID-2019. Nature 2020 [Epub ahead of print]. doi: 10.1038/s41586-020-2196-x. 32235945

[5] Ai T , Yang Z , Hou H , et al. Correlation of chest CT and RT-PCR testing in coronavirus disease 2019 (COVID-19) in China: a report of 1,014 cases. Radiology 2020 [Epub ahead of print]. doi: 10.1148/radiol.2020200642. PMC7233399 32101510

[6] Xu J , Wu R , Huang W , et al. Computed tomographic imaging of 3 patients with coronavirus disease 2019 with negative virus real-time reverse-transcriptase polymerase chain reaction test. Clin Infect Dis 2020 [Epub ahead of print]. doi: 10.1093/cid/ciaa207. PMC7184489 32232429

[7] Sun Y , Koh V , Marimuthu K , et al. Epidemiologic and clinical predictors of COVID-19. Clin Infect Dis 2020 [Epub ahead of print]. doi: 10.1093/cid/ciaa322. PMC7542554 32211755

[8] Interim guidelines for collecting, handling, and testing clinical specimens for persons for coronavirus disease 2019 (COVID-19). Centers for Disease Control and Prevention website.https://www.cdc.gov/coronavirus/2019-ncov/lab/guidelines-clinical-specimens.html. Updated April 14, 2020. Accessed April 16, 2020.

[9] Guo L , Ren L , Yang S , et al. Profiling early humoral response to diagnose novel coronavirus disease (COVID-19). Clin Infect Dis 2020 [Epub ahead of print]. doi: 10.1093/cid/ciaa310. PMC7184472 32198501

